# The Effect of Physiotherapy Interventions in the Workplace through Active Micro-Break Activities for Employees with Standing and Sedentary Work

**DOI:** 10.3390/healthcare10102073

**Published:** 2022-10-18

**Authors:** Stergios Vitoulas, Vasileios Konstantis, Irene Drizi, Sotiria Vrouva, George A. Koumantakis, Vasiliki Sakellari

**Affiliations:** 1 Physiotherapy Department, School of Health and Care Sciences, University of West Attica (UNIWA), 12243 Athens, Greece; 2 Laboratory of Advanced Physiotherapy (LAdPhys), Physiotherapy Department, School of Health and Care Sciences, University of West Attica (UNIWA), 12243 Athens, Greece; 3 Laboratory of Neuromuscular and Cardiovascular Study of Motion (LANECASM), Physiotherapy Department, School of Health and Care Sciences, University of West Attica (UNIWA), 12243 Athens, Greece

**Keywords:** work-related musculoskeletal disorders, ergonomics, injury prevention, exercise, counseling, micro-break activities, musculoskeletal pain

## Abstract

Workers worldwide experience a range of occupational musculoskeletal disorders that affect both the functionality of many parts of their body and their overall performance. Physiotherapists provide counseling and treatment programs during work. Recently, physiotherapy interventions have been introduced during work breaks. This study aimed to investigate the value of different types of workplace-based exercise programs administered during work breaks and compare them with counseling methods. Electronic searches were performed in relevant databases by keywords such as: workplace, musculoskeletal disorders, sedentary, standing, employees, micro-breaks, exercise interventions, and ergonomics. Initially, 706 articles were identified. An article sorting procedure was employed by two independent researchers, based on the inclusion and exclusion criteria set for this study, and after the removal of non-relevant articles (*n* = 391) or duplicates (*n* = 300), 15 randomized controlled trials (RCTs) remained for qualitative analysis. The methodological quality of the 13 RCTs was performed using the PEDro scale. No risk of bias evaluation was made. The findings suggested that active micro-breaks that contained various exercise programs including stretching, strengthening, torso stabilization, and ergonomic interventions were more beneficial than passive micro-breaks, reducing pain and the feeling of fatigue and increasing employees’ mood. It is concluded that micro-breaks are beneficial to employees with either orthostatic or sedentary work.

## 1. Introduction

Work-related musculoskeletal disorders (WR-MSDs) are the leading cause of injury, absenteeism, and reduced productivity [1]. A variety of professions with different body loading, movement, and external load management requirements are at risk of developing neuromusculoskeletal discomfort, pain, and disability in relation to the workers’ ability to manage these occupational-related demands [2,3,4]. Multiple symptoms, such as discomfort, paresthesia, tiredness, and restricted range of motion have been reported to be related to occupational demands [3]. Hazards in the workplace can be physical, psychological, social, or biomechanical [4]. Repetitive motion, forceful exertions, awkward postures, compression, and mechanical vibration are the key kinetic factors linked to MSDs [2,3,4,5].

The incidence of MSDs may decrease with accurate epidemiological knowledge, examination of ergonomic risks and musculoskeletal symptoms, and preparatory or compensatory workplace exercise undertaken at the beginning, during, or at the end of the working day, respectively [6]. Due to the interruption of work activities for exercise, compensatory workplace exercise is often referred to as a ‘short active break’ [6]. These pauses are intended to help relieve stress on the musculoskeletal system (muscles and joints) brought on by factors related to the work activities being performed and to correct for unnatural postures [6,7].

Employers and employees are currently trying to reduce the incidence of MSDs, absenteeism from work, and associated costs with the aid of health professionals, such as physiotherapists [5,6]. Workplace-based exercise programs target the neuromusculoskeletal system with the application of resistance, endurance, coordination, balance, postural re-education, and flexibility/stretching exercises [6]. To date, scientists have examined the effectiveness of a variety of programs that involve either exercise or passive breaks in different occupational settings or various ergonomic interventions, either for prolonged standing or for sitting positions [7,8,9]. A previously conducted systematic review examining the effectiveness of interventions in the workplace for the prevention of upper extremity musculoskeletal disorders reported strong evidence for resistance training, and moderate evidence for stretching programs [7]. Another systematic review examined the benefit of interrupting work in sitting via standing or walking interventions, reporting in general no effect in the reduction of musculoskeletal complaints [8]. A third study examined the efficacy of various work-break schedules for reducing work-related musculoskeletal complaints and disorders in healthy employees when compared to traditional or alternative work-break schedules, concluding that different work-break frequencies and types may not substantially reduce the incidence of musculoskeletal disorders [9]. However, micro-breaks are important not only from a physical but also from a psychological perspective, such as fear of movement, depression, anxiety [9,10], therefore additional outcomes may be required to assess the benefit of such interventions.

Determining the optimal type of break, frequency, and duration to be integrated into the work program could prevent possible occupational injuries without interfering with the work process. Previous systematic reviews either dealt with an array of workplace interventions (exercise studies included) involving upper limb disorders and symptomatology [7] or were concerned with more specific interventions (standing or walking) in sedentary occupations [8]. A third study involved both active and passive micro-break interventions [9]. However, the last two mentioned systematic reviews included studies with a high risk of bias [8,9]. Adherence is of importance in all such interventions and having a wearable device may act as a reminder as well as an incentive to maintain a good physical activity level and reduce sedentariness [11]. The aim of this study was to qualitatively analyze the effects of workplace-based active micro-breaks in the form of exercises targeting the health improvement (pain, disability, muscle performance characteristics, quality of life) delivered in a variety of work environments in studies of moderate-to-high methodological quality.

## 2. Methods

### 2.1. Search Strategy

The search strategy was performed using the PICO method to define the research query, while the MeSH (Medical Subject Headings) terms used in the search in the various databases were: workplace, musculoskeletal disorders, sedentary, standing, employees, micro-breaks, exercise interventions, ergonomics, pain, disability, and randomized controlled trials (RCTs).

Electronic searches were performed using the following databases: PubMed, EMBASE, PsycINFO, and ResearchGate. An article-sorting procedure was then performed. Then, each of the two researchers read the title, and the abstract of all studies, and assessed their eligibility according to the inclusion and exclusion criteria (Table 1). Consequently, the eligible articles were reviewed by two independent researchers, after the non-eligible and duplicates were removed. Only RCTs of methodological quality ≥6 on the PEDro scale were included in this review (Table 2).

### 2.2. Study Design

The current study is a narrative review of the literature. A criterion of inclusion for the articles was a score on the PEDro scale ≥6. A total of 15 collected articles [12,13,14,15,16,17,18,19,20,21,22,23,24,25,26], considered moderate-to-high quality, fulfilled this inclusion criterion. 

### 2.3. Statistical Analysis

**Figure 1:**
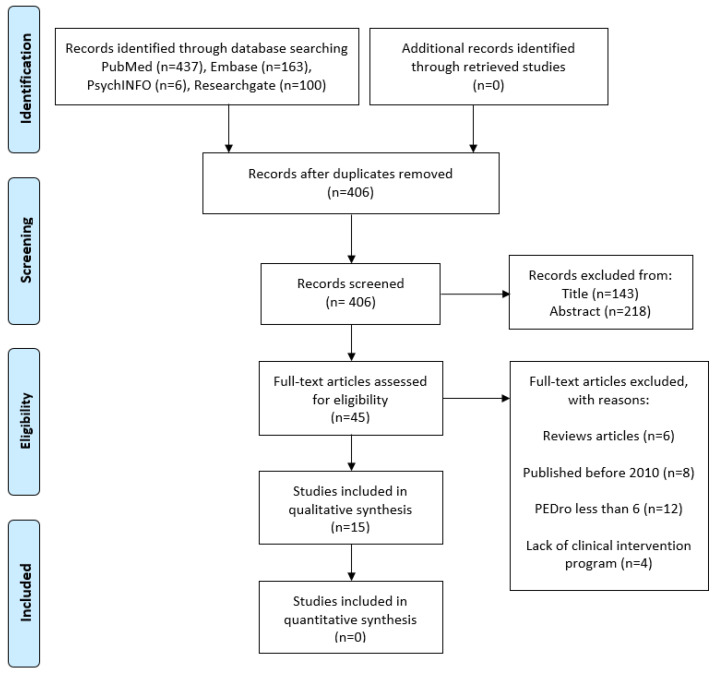
PRISMA flowchart showing the selection procedure for the studies in this review.

This was a narrative review, which consisted of all the essential steps of a systematic review, without proceeding to an analysis and synthesis of quantitative data, such as risk ratio, odds ratio, and mean-standard deviations of every study included. The risk of bias of the included studies as well as the possible causes of heterogeneity between the results of the studies were not assessed. The number of all articles initially identified, assessed, sequentially excluded (with reasons), and those finally included in the study were presented in a flowchart that was created according to the PRISMA checklist used in the systematic review for the recording and documentation of the bibliographic search (Figure 1). Finally, 15 articles were identified as suitable and were analyzed in the review.

## 3. Results

### 3.1. Characteristics of the Included Studies

The characteristics of the included studies are analytically presented in the Supplementary Material (Table S1). In summary, the studies included a variety of occupations with participants working either in a standing or in a sitting position. The population of included studies ranged from 30 to 537 participants. The included interventions either targeted muscle strength or stretching of key muscle groups, or a more generalized combination of strength, stretching, and joint mobility. The duration of the exercise programs generally varied between 4 and 20 weeks [12,13,14,15,16,17,20,22,23,25,26]; however, there was one study that observed participants in 5 equidistant time intervals included within 1 h [24], one at 2 h [21], one at 6 months [19], and another that included a 6- and a 12-month follow-up [18]. Three of the included studies had exclusively recruited female participants, due to the nature of the occupation studied. A range of outcome measures has been utilized (pain intensity or discomfort, pain interference, fatigue, isometric muscle strength of various muscle groups, aerobic fitness, electromyographic activity, productivity, disability, and work-related factors). In general, active exercise breaks help reduce physical pain and fatigue and improve the mood of employees. 

### 3.2. Strengthening Programs in Standing Occupations

The implementation of different designs of resistance training in the workplace showed a reduction in pain intensity in most studies [12,13,14,15,16,17]. A significant reduction in lumbar pain was observed after intense kettlebell training for 8 weeks (reduction by 57%) in the study of Jay et al. [12], while the contribution of a strengthening program for pain relief among industrial workers of Zebis et al. [13] was significant in the reduction of pain in the intervention group in the areas of the shoulder and neck. Another study by Jakobsen et al. [14] observed a decrease in the lumbar pain intensity with a work-based 10-week exercise program applied in only female healthcare workers. Sundstrup et al. [15] observed a significant improvement in the Work Ability Index (WAI) among slaughterhouse workers, following a 10-week exercise program. Muñoz Poblete et al. [16] explored the effectiveness of a workplace-based muscle resistance training exercise program for the intervention group and a mild stretching program for the control group and concluded that there was a reduction in upper extremity pain after 16 weeks of intervention in favor of the intervention group. Furthermore, Muñoz Poblete et al. [16] found that individuals can benefit from a set of exercises for about 10 to 20 min a day and at least 3 times a week, by adapting strengthening programs to employees’ available time. In addition, Andersen et al. [17] found that as little as 2 min of daily progressive resistance training for 10 weeks results in clinically relevant reductions of pain and tenderness in healthy adults with frequent neck/shoulder symptoms.

Specifically, in the study of Jay et al. [12], the strength training increased participants’ extensor muscle strength levels in the lumbar spine (mainly those used for lifting), while it did not appear to be effective for the torso flexor muscle groups and the shoulder area. Sundstrup et al. [15] also demonstrated an increase of the muscular strength of the wrist and the upper limb, with an increase of the maximum isometric contraction of the grip in the intervention group. Evidence of statistically significant strengthening in the lumbar muscles was observed in the study of Jakobsen et al. [14] for the workplace-based intervention group. Finally, in the research study of Zebis et al. [13] there were no clear effects of the program on the muscular strength of the included participants.

Another outcome studied was the fatigue and aerobic capacity of the individuals. Sundstrup et al. [15] observed that after the end of the strengthening program there was a significant increase in the time to fatigue in the intervention group, an improvement rate approaching 97%, while there was no change in the control group, and a parallel increase in the WAI. Aerobic capacity was also studied as an outcome by Jay et al. [12], but with no clear effect of the intervention. The studies of Zebis et al. [13] and Jakobsen et al. [14] did not include aerobic capacity as an outcome.

### 3.3. Home-Based Exercise versus Workplace Exercises in Standing Occupations

The results of Jakobsen et al. [14] were not in favor of home training for healthcare workers, as the workplace-based intervention group excelled in all tested parameters. The intensity of pain was found to decrease in the work-based group more than the home-based group both in the lumbar region and in the shoulder and neck areas. The decrease in pain intensity was observed in 78% of the participants in the work-based intervention group. The intensity of pain decreased in 42% of the participants for the home-based physical exercise group. In addition, muscle strength was found to be higher in the work-based group. Furthermore, the study considered the workplace as the most suitable for increasing mood compared to physical exercise at home.

### 3.4. Relaxation Exercise, Stress Reduction, and Ergonomic Interventions in Standing Occupations

Taulaniemi et al. [18], in a secondary analysis of an RCT, examined exercises that aimed at relaxation and reduction of stress from a neurophysiological perspective. This research tested a workplace exercise program involving female healthcare workers with nursing duties. After 6 and 12 months, the mean reduction in pain in the exercise group was greater compared to the non-exercise group, while in further analysis, the difference in pain reduction was greater in the exercise group in relation to the control group. Furthermore, the program improved lumbar mobility, maintained or even increased abdominal muscle strength, and showed that the trainees in the intervention group perceived less feelings of fatigue and had better recovery for work.

The study by Sundstrup et al. [15] compared a workplace strengthening exercise program with a standard ergonomic intervention. The resulting effect of the ergonomic intervention on fatigue, pain intensity, and muscle strengthening were a clear reduction.

### 3.5. General Programs of Physical Activities in Standing Occupations

Rasotto et al. [19] applied a tailored exercise program composed of exercises for strengthening, stretching, and mobility of body parts in a female working population suffering from musculoskeletal disorders. The results of this research show a reduction in pain in the intervention group. In addition, the grip strength and the strength in the shoulder area muscles increased by 4.9% and 70.6%, respectively. A study by Gram et al. [20] investigated the effect of the combination of aerobic exercise and strengthening as individually tailored exercise programs for male construction workers. They observed a statistically significant increase in maximal aerobic capacity compared to the group that attended the exercise sessions. The study demonstrated good effectiveness for integrating short exercise bouts of 20 min, 3 times a week, into organizational routines among construction workers.

### 3.6. Effect of Stretching in Sitting Occupations

Six articles studying sedentary occupations were selected as representative of this group. Ding et al. [21], Caputo et al. [22], Mehrparvar et al. [23], Nakphet et al. [24], and Lacaze et al. [25] studied the stretching exercise applied to at least one intervention group, either solely or in combination with another form of activity. In another study by Santos et al. [26], a stretching program was followed by the control group and not the intervention group. In four of the six articles above, musculoskeletal discomfort is mentioned as the main outcome measure. In the study by Lacaze et al. [23], an exercise program consisting of stretching and joint mobilization was applied to call-center operators, which resulted in a significant reduction in post-intervention discomfort by 6.5 points, with the intervention group showing the most statistically significant reduction in the incidence of neck and shoulder discomfort, and discomfort of the spine and buttocks. In the upper and lower extremities, the reduction of discomfort was similar between the two groups.

The results of Ding et al. [21] showed that the most significant relief in terms of perceived discomfort from prolonged sitting work was accomplished by the standing and stretching group for 5 min. Caputo et al. [22] attempted to examine the effectiveness of group resistance work exercises, specifically in the neck and shoulder area in video display unit workers (VDU). Mehrparvar et al. [23] did not find a significant difference between the intervention groups. In the latest study of Nakphet et al. [24], all VDU unit operators in an intervention group showed reduced musculoskeletal discomfort in all parts of the body immediately after the break than at the end of each 20-min work period.

Regarding the effect of stretching on fatigue, the study by Ding et al. [21] included interesting data, where the 5-min standing and stretching group had the best outcome, with a significant reduction in fatigue levels, maintaining the muscles in a non-fatigued state for 30–45 min. The exercise group with a stretching, mobilization, and relaxation program had a positive effect on mental fatigue in the study of Lacaze et al. [25]. In particular, the exercise group appeared to have better results in employee memory and fatigue with fewer speech errors compared to the control group. Santos et al. [26] did not detect a significant difference between the progressive resistance exercise group and the stretching and stretching exercise group. Similarly, Nakphet et al. [24] found no significant difference from the comparison of the stretching and dynamic contraction groups for muscle fatigue. They concluded, however, that any form of activity during the break is beneficial in preventing neck and shoulder fatigue.

According to Mehrparvar et al. [23], both the groups with ergonomic modification of the workstation and the one with workplace exercises, including stretching, reduced their musculoskeletal pain in a similar way, except for lumbar pain. Stretching in the intervention group reduced lower back pain more than in the ergonomic group. In the study of Caputo et al. [22] pain and pain-related chronic neck pain disability decreased similarly and without significant differences between the neck-shoulder resistance exercise group and the conventional stretching and postural exercise group.

## 4. Discussion

Most of the strengthening programs employed in the three studies [12,13,15,16] were performed 3 times a week, with an average duration of 20 min per session. It is known that the muscles are strengthened more sufficiently when they undergo dynamic and progressive training with concentric and eccentric high-intensity contractions and with a maximum of 8 to 12 contractions using dumbbells, elastic bands, and anti-gravity exercises [27]. Studies, such as the ones of Jay et al. [12] and Zebis et al. [13], found statistically significant reductions in the pain of shoulder and neck areas compared to the control group. Muñoz Poblete et al. [16] concluded that there was a reduction in upper extremity pain after 16 weeks of a set of exercises for about 10 to 20 min/day and at least 3 times/week, in favor of the strengthening group. Finally, Andersen et al. [17] found that as little as 2 min of daily progressive resistance training for 10 weeks results in clinically relevant reductions of pain in adults with frequent neck/shoulder symptoms. Other studies [12,14] found that lumbar pain was significantly reduced compared with the control group.

Kettlebell exercises focused on the upper extremities and lumbar region have been shown to have a positive effect on combating pain [12]. It is reported that generally, in a home program, it is difficult to perform exercises using equipment such as kettlebells [12] or other special resistance bands, as a supervisor is constantly needed to give advice and watch out for injuries [14]. Thus, providing videos or posters depicting the exercises is an affordable financial solution, which, however, cannot replace the trainer himself. According to Jakobsen et al. [14], exercise in a familiar environment does not have a positive effect on employees, which highlights the importance of the concept of exercise in the workplace.

The contribution of stretching and mobilization exercises to pain intensity is not clearly supported by the current literature [21,22,23,24,25,26]. The benefits of stretching may be mainly related to its recovery benefits. Still, studies such the one by da Costa and Vieira [28] emphasize the need to use stretching programs in the workplace accompanied by other types of exercise, emphasizing that it cannot stand alone as a treatment method to reduce work-related musculoskeletal disorders.

Strengthening exercises are time-consuming and highly individualized, whereas micro-break activities can last for about 2 min. Nevertheless, the inclusion of stretching exercises between breaks in working hours is feasible despite its practical difficulties. For instance, the time needed to complete the exercise process may extend the duration of the micro-break. Based on the American College of Sports Medicine, which refers to the guidance for prescribing exercise [27], the employee needs a stretching activity of 10–30 s and 3–5 repetitions, with the total time of the whole procedure lasting a little more than the normal duration of a short break, in order to gain the benefits of the stretching. Active stretching normally does not cause additional compression or tension to the tissues and joints, as it is based on voluntary movement resulting in the restoration of extracellular matrix homeostasis and normal arrangement of tissue structures [28]. Therefore, the classic form of static stretching cannot meet the requirements of rehabilitation and prevention of musculoskeletal injuries in standing occupations [29], while additional movement promotes the healing process more easily.

Methods such as the No Lifting Policy need to be further analyzed in the modern professional world, as it involves financial issues as well [30]. There is a need for further study of interval exercises and ergonomic intervention in the workplace, through well-designed studies. Another important emerging method used in order to return to or remain at work for workers with chronic musculoskeletal condition, is a personalized self-management program that contains a set of psycho-educational techniques based on behavioral and cognitive education [31]. A commonly accepted program of short break workouts has not yet been formulated for sitting professionals. According to Jepsen and Thompsen [32], stretching alone cannot be a method of preventing disorders for people working in computer workstations. A recent review came to a similar statement, concluding that both stretching exercises and ergonomic intervention have not shown satisfactory results on their own, in terms of work-related musculoskeletal pain and discomfort [33]. Therefore, the combination of resistance/strengthening exercise with stretching and/or ergonomic intervention in the workplace is recommended.

Ylinen et al. [34] observed that isometric strength training and dynamic endurance training effectively reduced chronic neck pain and disability, while aerobic exercise and stretching alone, performed by the control group, proved to be much less beneficial. Stretching leads to several benefits, such as reducing discomfort caused by prolonged sedentary behavior [35], reducing pressure on the intervertebral discs [36], removal of lactic acid, increase in blood circulation, and stimulation and alertness of workers [37,38]. Stretching can also increase the range of motion and reduce the pain [39,40]. An important point of attention that should not be omitted in respect to stretching and resistance/strengthening exercises is the inclusion of warm-up time [41].

Regarding active and passive breaks, Ding et al. [21] stated that active cessation of work in standing and stretching for 5 min was significantly more beneficial than passive breaks on the chair. On the other hand, Nakphet et al. [24] stated that there is no significant difference between active and passive breaks in the sedentary work environment.

In contrast to stretching, resistance/strength training has been studied extensively, with most results being supportive [12,13,14,15,16,17,26]. Among the types of exercise proposed are strength training [34,40,41], endurance training [34,41], and muscle coordination training [41] to alleviate pain. However, the most appropriate type of exercise has not yet been identified. Tomanova et al. [42] suggest stabilization exercises as well as stretching and relaxation for effective treatment of lower back pain. Sipaviciene and Kliziene [43] compared the lumbar stabilization exercise with the lumbar muscle strengthening exercise in patients who perform sedentary work, with the group that activated the deep stabilizers of the trunk and received pelvic control training presenting the best results in terms of pain and disability.

Two other important factors that need to be identified to determine the most appropriate type of break are the frequency and duration of the short breaks. Regarding the frequency, there is a discrepancy between the surveys with the short breaks varying from 10 s to 15 min every 6 min and 2 working hours, respectively. Vijendren et al. [44] suggested micro breaks of 20 s to 30 s every 20 min to 30 min of work. As reported, a short program of stretching exercises every 30 min reduced musculoskeletal discomfort [45], while a corresponding stretching program every 15 min reduced discomfort, eye strain, and shoulder strain [46]. A similar program of frequent cessation of activity for 5 min every 30 min of work improved the productivity of employees [47].

Ultimately, in terms of productivity, there is no evidence that general micro-breaks have a detrimental effect on working productivity, as older studies have implied [48], and which has not been recently verified [9].

## 5. Conclusions

Breaks and micro-breaks were found to be applicable to employees during standing and sitting occupations. Active breaks with a specified exercise program were more beneficial than passive breaks. Exercise in the workplace is recommended over exercise at home or in places other than work. Short breaks are recommended for at least 10 to 15 min every 40 to 60 min of continuous work, otherwise it would be preferable to take a break of 3 or 5 min every 30 min, regardless of the lunch break.

In standing occupations, active stretching and strengthening exercises are the two predominant types of exercises used for the prevention and rehabilitation of musculoskeletal injuries during work breaks. In addition, the most preferable exercise for sedentary occupations is that of stretching, followed by resistance and strengthening exercise. When the worker is exposed to prolonged sedentary postures, the lumbar spine, the neck, shoulder, upper extremities, wrist, and the back are mainly affected. Thus, exercise that has a preventive and therapeutic role puts emphasis on these areas. Ideally, an active workout/micro-break program in the workplace that includes stretching, strengthening, torso stabilization as well as ergonomic interventions in the workplace, should focus on the needs of each profession.

## Figures and Tables

**Table 1:** Inclusion and exclusion criteria.

Inclusion Criteria	Exclusion Criteria
Employees as participants	Non-employees as participants
Active employee breaks	Exclusively passive breaks
Published from January 2010 onwards	Published until December 2009
At least a 6/10 score on the PEDro scale	Less than a 6/10 score on the PEDro scale
Sufficient number of participants per treatment group	Less than 15 participants per treatment group
Randomized controlled trials	Non-randomized controlled trials

**Table 2:** Evaluation of articles using the Physiotherapy Evidence Database (PEDro) scale score.

	Items										Total Score
Author, Publication Date	1	2	3	4	5	6	7	8	9	10	
Jay et al., 2011 [12]	X		X			X	X		X	X	6/10
Zebis et al., 2011 [13]	X	X	X					X	X	X	6/10
Jakobsen et al., 2015 [14]	X		X			X	X	X	X	X	7/10
Sundstrup et al., 2014 [15]	X	X	X			X	X	X	X	X	8/10
Muñoz Poblete et al., 2019 [16]	Χ	Χ				Χ	Χ		Χ	Χ	6/10
Andersen et al. 2011 [17]	X	X	X			X	X	X	X	X	8/10
Taulaniemi et al., 2019 [18]	X	X	X			X		X	X	X	7/10
Rasotto et al., 2015 [19]	X	X	X			X		X	X	X	7/10
Gram et al., 2014 [20]	X		X				X	X	X	X	6/10
Ding et al., 2020 [21]	X	X	X	X			X	X	X		7/10
Caputo et al., 2017 [22]	X	X	X			X		X	X	X	7/10
Mehrparvar et al., 2014 [23]	X	X	X			X		X	X	X	7/10
Nakphet et al., 2014 [24]	X	X	X	X			X	X	X		7/10
Lacaze et al., 2010 [25]	X	X	X	X		X	X	X	X	X	9/10
Santos et al., 2020 [26]	X			X		X	X	X	X	X	7/10

Items correspond to the following criteria, 1: random allocation, 2: concealed allocation, 3: baseline comparability, 4: blind subjects, 5: blind therapists, 6: blind assessors, 7: adequate follow up, 8: management as planned or intention-to-treat analysis, 9: between-group comparisons, 10: point estimates and variability.

## Data Availability

Not applicable.

## Supplementary Materials

The following supporting information can be downloaded at: https://www.mdpi.com/article/10.3390/healthcare10102073/s1, Table S1: Details of included studies.
