# Supplementary material for: The Effect of Physiotherapy Interventions in the Workplace through Active Micro-Break Activities for Employees with Standing and Sedentary Work
Source: Healthcare (Basel). 2022 Oct 18;10(10):2073. doi: 10.3390/healthcare10102073 (PMC9603092; doi:10.3390/healthcare10102073)
Supplement: Supplementary file 1 [file healthcare-10-02073-s001.zip › healthcare-1925690-supplementary.pdf]

Table S1. Details of included studies.

Author, Publication Date	Aim	Sample Methods	Intervention Groups	Outcomes	Results
**Lacaze et al., 2010**	Evaluation of musculoskeletal discomfort and mental and physical fatigue in the call-center workers of an airline company before and after a supervised exercise program compared with rest breaks during the work shift	64 call-center operators<br><br>RCT	Experimental group (n=32): 10-min daily exercise session for 2 months.<br><br>Control group: 10-min daily rest break during the same period.	Each subject was evaluated once a week by means of:<br><br>The Corlett-Bishop body map with a visual analog discomfort scale and The Chalder fatigue questionnaire<br><br>at 8 weeks	• Musculoskeletal discomfort decreased in both groups, but the reduction was only statistically significant for the spine and buttocks (p=0.04) and the sum of the segments (p=0.01) in the experimental group.<br>• The experimental group showed significant differences in the level of mental fatigue, especially in questions related to memory Rienzo, #181ff and tiredness (p=0.001)
**Andersen et al., 2011**	Evaluation of the effectiveness of small daily amounts of progressive resistance training for relieving neck/shoulder pain results in clinically relevant reductions of pain and tenderness in healthy adults with frequent neck/shoulder symptoms	198 healthy white-collar employees working at least 30h per week and with frequent neck/shoulder pain<br><br>RCT	Experimental group 1 (n=66): Resistance training with elastic tubing for 2 minutes per day 5 times per week<br><br>Experimental group 2 (n=66): Resistance training with elastic tubing for 12 minutes per day 5 times per week<br><br>Control group (n=66): Weekly information on general health	Primary outcomes:<br><br>Intensity of neck/shoulder pain<br><br>Examiner-verified tenderness of the neck/shoulder muscles<br><br>Isometric muscle strength<br><br>at 10 weeks	• Compared with the control group, neck/shoulder pain and tenderness, respectively, decreased 1.4 points (95% confidence interval -2.0 to -0.7, p < 0.0001) and 4.2 points (95% confidence interval -5.7 to -2.7, p < 0.0001) in the 2-minute group and 1.9 points (95% confidence interval -2.5 to -1.2, p < 0.0001) and 4.4 points (95% confidence interval -5.9 to -2.9, p < 0.0001) in the 12-minute group.<br>• Compared with the control group, muscle strength increased 2.0 Nm (95% confidence interval 0.5 to 3.5 Nm, p = 0.01) in the 2-minute group and 1.7 Nm

					(95% confidence interval 0.2 to 3.3 Nm, p = 0.02) in the 12-minute group.
**Jay et al., 2011**	Worksite intervention using kettlebell training to improve musculoskeletal and cardiovascular health	40 adults from occupations with a high prevalence of reported musculoskeletal pain symptoms<br><br>Single blind RCT	Training group (n=20): Ballistic full-body kettlebell exercise 3 times per week for 8 weeks<br><br>Control group (n=20)	Pain intensity (neck/shoulders and low back)<br><br>Isometric muscle strength, and<br><br>Aerobic fitness<br><br>at 8 weeks	In training group, compared with the control group:<br>• Pain intensity of neck/shoulders decreased 2.1 points [95% confidence interval (95% CI) -3.7- -0.4] and pain intensity of the low back decreased 1.4 points (95% CI -2.7- -0.02)<br>• Increased muscle strength of the trunk extensors (P<0.001), but not of the trunk flexors and shoulders.<br>• Aerobic fitness remained unchanged.
**Zebis et al., 2011**	Evaluation of the effect of implementing strength training at the workplace on non-specific neck and shoulder pain among industrial workers	537 adults from industrial production units (occupations with high prevalence of neck and shoulder pain)<br><br>Cluster-RCT	Experimental group (n=282): 20 weeks of high-intensity strength training for the neck and shoulders three times a week<br><br>Control group (n = 255): advice to stay physically active.<br>The strength training program followed principles of progressive overload and periodization.	Primary outcome: changes in self-reported neck and shoulder pain intensity (scale 0-9)<br><br>at 20 weeks	• In the training group compared with the control group, neck pain intensity decreased significantly (-0.6, 95% CI -1.0 to -0.1) and shoulder pain intensity tended to decrease (-0.2, 95% CI -0.5 to 0.1, P = 0.07).<br><br>• For pain-cases at baseline (pain intensity > = 3) the odds ratio - in the training group compared with the control group--for being a non-case at follow-up (pain intensity < 3) was 2.0 (95% CI 1.0 to 4.2) for the neck and 3.9 (95% CI 1.7 to 9.4) for the shoulders.

**Gram et al., 2014**	To assess the physical capacity of construction workers and evaluate the effect of individually tailored exercise programs on their physical fitness and muscular capacity	67 male constructions workers<br><br>RCT	Exercise group (n=35): Intervention lasted 12 weeks, and the exercise group trained 3 x 20 minutes a week. Data from the first health check were used to tailor the exercise in the interventions.<br><br>Control group (n=32): 1-hour lecture on general health promotion.	VO2 max<br><br>Body mass<br><br>Muscle Strength<br><br>Blood pressure<br><br>Cholesterol levels<br><br>at 12 weeks	• With respect to the intervention, group x time analyses showed a significant difference in estimated change in VO(2max)of 0.4 l/min for the exercise group and 0.0 l/min for the control group (P<0.001).<br>• Body mass and other general health measures remained unchanged.
**Mehrparvar et al., 2014**	Comparison of the effect of ergonomic modifications and workplace exercises on musculoskeletal pain and discomfort in a group of office workers	164 office workers<br><br>RCT	Ergonomic modification (n=83): correcting the arrangement of workstation and changing some equipment<br><br>Workplace exercises (n=81): Stretching exercises focusing on neck, shoulders, low back, and hand and wrist	Musculoskeletal complaints compared before and after 1 month interventions<br><br>at 4 weeks	• Both interventions significantly reduced complaints in a similar manner except for low back pain which was reduced in exercise group more than the other group.
**Nakphet et al., 2014**	Evaluation of different types of activities during rest-break interventions among symptomatic video display unit (VDU) operators performing	30 symptomatic VDU operators<br><br>RCT	Active break group 1 (n=10): Stretching<br><br>Active break group 2 (n=10): Dynamic movements	Neck and shoulder muscle activity<br><br>Muscle discomfort (Borg's CR-10 scale)	• No significant differences between the types of activities during breaks on neck and shoulder muscle activity, muscle discomfort or productivity.<br>• Significant difference in the level of muscle discomfort over time.

	prolonged computer terminal work		Control group (n=10)<br><br>The subjects performed the same typing task for 60 min and received 3-min breaks after each 20 min of work.	Productivity (counting words)<br><br>at 1 hour	
**Sundstrup et al., 2014**	To evaluate the effect of two contrasting interventions on work ability among slaughterhouse workers with chronic pain and work disability	66 slaughterhouse workers with upper-limb chronic pain and work disability<br><br>RCT	Strength training: 10 weeks of either for the shoulder, arm, and hand muscles (3 times per week, 10 minutes per session)<br><br>Ergonomic training: usual care control group	Work ability index (WAI)<br><br>10 weeks	• Compared with the ergonomic training group, WAI increased 2.3 [95% confidence interval (95% CI) 0.9–3.7] in the strength training group corresponding to a moderate effect size (Cohen's d 0.52).<br>• Within-group changes indicated that between-group differences were mainly caused by a reduction in WAI in the ergonomic group. Of the 7 items of WAI, item 2 (work ability in relation to the demands of the job) and item 7 (mental resources) increased following strength training compared with ergonomic training (P<0.05).
**Jakobsen et al., 2015**	Investigation of workplace versus home-based physical exercise on musculoskeletal pain among healthcare workers	200 female healthcare workers from 18 departments at 3 hospitals.<br><br>RCT	10 weeks of:<br>(i) workplace physical exercise (WORK) performed during working hours for 5×10 minutes per week and up to 5 group-based coaching sessions on motivation for regular physical exercise	Primary outcome: Average pain intensity (0-10 scale) in the low back and neck/shoulders<br><br>at 10 weeks	• Pain intensity, back muscle strength and use of analgesics improved more following WORK than HOME (P<0.05).<br>• Between-group differences at follow-up (WORK versus HOME) was -0.7 points for pain intensity [95% confidence interval (95% CI) -1.0- -0.3], 5.5 Nm for back muscle strength (95% CI 2.0-9.0), and -0.4 days per week for use of analgesics (95% CI -0.7- -0.2).

			(ii) home-based physical exercise (HOME) performed during leisure time for 5×10 minutes per week.<br><br>Both groups received ergonomic counseling on patient handling and use of lifting aides.		• The effect size for between-group differences in pain intensity was small (Cohen's d=0.31).
**Rasotto et al., 2015**	Evaluation of a tailored physical activity protocol performed in a work environment with a group of female workers employed in manual precision tasks to reduce upper limb pain	60 female workers<br><br>RCT	Intervention group (n=30): 6-month, twice-a-week, tailored exercise program<br><br>Control group (n=30): no intervention.	Pain<br><br>ROM<br><br>Grip strength<br><br>at 6 months	• The IG showed a reduction on shoulder pain accompanied by increases on the range of motion measures. In addition, reductions in upper limb pain and neck disability were detected with concomitant increases in grip strength.
**Caputo et al., 2017**	Evaluation of the efficacy of group-based neck-shoulder resistance exercises on symptoms and muscular function in VDU workers with W-RCNP.<br>NSRE and SPE are feasible and equally effective in reducing pain and pain-related disability as group exercises in W-RCNP	35 employees with CNP<br><br>RCT	Neck-shoulder resistance exercise (NSRE) group or to conventional stretching and postural exercise (SPE) group. Participants trained 45 min, twice a week for 7 weeks.	Primary outcomes: Pain intensity (0-10 numeric rating scale, NRS) and Pain-related disability (Vernon Neck Disability Index, NDI).<br><br>Secondary outcomes: Cervical active range of motion (AROM), endurance of neck flexors, strength of shoulder abductors, and	• Post-treatment, pain and disability decreased 2.5 and 5 points in NSRE (p = 0.001) and 5 and 8 points in SPE (p = 0.002), with no significant differences between groups.<br>• AROM improved significantly in both groups; between-group differences were not significant.<br>• Neck flexors endurance and shoulder abductors strength improved by 59.6 and 34.2% (p = 0.001) in the NSRE group and by 27.6% (p = 0.009) and 2.3% (p = 0.002) in the SPE group; between-group

				health-related quality of life (SF-36).<br>at 7 weeks	differences were significant (p = 0.016 and p < 0.001, respectively). SF-36 score remained unchanged.
**Taulaniemi et al., 2019**	Examination of 6 months of neuromuscular exercise among female healthcare personnel with sub-acute or recurrent low back pain	291 female healthcare workers with non-specific LBP<br><br>RCT	Exercise group (n=57)<br><br>Counselling group (n=53)<br><br>Combined exercise and counselling group (n=55)<br><br>Control (n=54)<br><br>Exercise was performed twice a week (60 min) in three progressive stages focusing on controlling the neutral spine posture.	Primary outcome: Pain Intensity<br><br>Pain interference<br>Lumbar movement control<br>Fitness<br>Work-related factors<br><br>6- and 12-months' follow-up	• The exercise intervention reduced pain (p = 0.047), and pain interfering with work (p = 0.046); improved lumbar movement control (p = 0.042), abdominal strength (p = 0.033) and physical functioning in heavy nursing duties (p = 0.007); but had no effect on other fitness and work-related measurements when compared to not exercising.<br>• High exercise compliance resulted in less pain and better lumbar movement control and walking test results.
**Muñoz Poblete et al., 2019**	Evaluation of a workplace-based muscle resistance training exercise program in the presence of pain and musculoskeletal dysfunction of the upper extremities	109 manufacturing workers exposed to repetitive movements and excessive effort in the workplace<br><br>RCT	Experimental group (n=53): Resistance-based exercise program<br><br>Control group (n=56): Stretching exercises	Perceived pain in upper limbs<br><br>at 16 weeks	• The muscle resistance training exercise had a protective effect on the intensity of pain perceived by workers in their upper limbs (RR: 0.62 95% CI 0.44-0.87) compared with the group of workers who performed stretching exercises.
**Ding et al., 2020**	This study aimed to explore the variation in	48 sedentary office workers	48 participants were divided into six groups to	EMG amplitudes and spectra	• Standing and stretching for 5 mins was the most effective break type, and this

	muscle activities during prolonged sitting work and when and how to take a break to mitigate the risk of muscle symptoms		compare the effectiveness of every break type (passive break, active break of changing their posture, and stand and stretch their body with 5 or 10 mins). Finally, data consisting of EMG amplitudes and spectra and subjective assessment of discomfort were analyzed.	Subjective assessment of discomfort<br><br>at 2 hours	type of break could keep the muscles' state at a recovery level for about 30−45 mins.
**Santos et al., 2020**	Evaluation of the effects of a resistance exercise program on perceived fatigue control among industrial workers	204 employees from the dairy industry<br><br>Cluster RCT	Intervention group (IG) (n = 98): Resistance exercises using progressively greater loads.<br><br>Control group (CG) (n = 106): General exercise using elastic bands.<br><br>The exercise protocols were performed three times per week for 20 min.	Primary outcomes: Perceived fatigue control (Need for Recovery Scale) and maximum muscle strength and one-repetition maximum contraction (1-RM)<br>Secondary outcomes: Musculoskeletal complaints<br>Physical activity level<br>Perceived risk factors<br>Physical fitness (BMI, vital signs, and body fat percentage)<br>Workers´ productivity<br><br>at 4 months	• The IG did not show to be superior to the CG, although both groups improved perceived fatigue control and muscle strength after the resistance physical exercise program in the worplace.<br>• There was also no significant difference between the groups for musculoskeletal complaints and other secondary variables analyzed.<br>• However, both groups showed significant improvements between baseline and after 4 months of intervention for all evaluated outcomes ($p < 0.05$).

RCT: randomized controlled trial, ROM: range of movement, VDU: video display unit, W-R CNP: work-related chronic neck pain
